# Avoiding Catastrophic Mutations Accurately Predicts Amino Acid to Codon Pairing

**DOI:** 10.1007/s00239-025-10294-0

**Published:** 2025-12-20

**Authors:** Peter Nonacs, Thomas Nonacs

**Affiliations:** 1 Department of Ecology and Evolutionary Biology, University of California, Los Angeles, CA 90095 USA; 2 Department of Entomology, University of California, Riverside, CA 92521 USA

**Keywords:** Genetic code, Mutations, Evolution, Codon networks, Amino acid, Biological sciences

## Abstract

DNA codon mutations involving Stop signals or the amino acid cysteine can be especially damaging. The former can break protein sequences or add extraneous amino acids. The latter can add or subtract disulfide bonds crucial in protein folding. We present a hypothetical scenario where Stop codons were present early in the evolution of the genetic code; and minimizing catastrophic mutations for code networks affected all subsequent amino acid/codon pairings. Predicted features of this “Catastrophic Mutation Minimization Hypothesis” (CMMH) are that: (1) Cysteine is mutationally adjacent to Stop, isolating a contiguous codon ‘neighborhood’ with high potential for catastrophe. (2) The sequence of amino acid additions order determines codon assignments through minimizing network-wide mutation costs. Overall, codon locations for 16 of the 20 amino acids in the genetic code are consistent with the CMMH, as are multiple other predictions. We propose an antecedent genetic code consisted of 16 doublet codons specifying 13–14 amino acids. Two variations of these networks are less susceptible to catastrophic mutations than 88.2–97.5% of randomly generated ones. Unlike some previous hypotheses, CMMH does not require the total replacement or rearrangement of amino acids at codons, with its disruptive potential for protein synthesis. Finally, the composition of this ancestral doublet genetic code has all the modern code’s utility: amino acids from four chemical types; start and stop signals; metal-binding ability; disulfide bridging for creating protein shapes; and possible epigenetic gene regulation. Thus, the modern code likely evolutionarily fine-tuned antecedent capabilities, rather than significantly increasing competence for making complex proteins.

**Supplementary Information:**

The online version contains supplementary material available at 10.1007/s00239-025-10294-0.

## Introduction

All living organisms, including viruses, basically share the same genetic code of 64 trinucleotide codons specifying 20 amino acids used for protein construction. There are several interrelated questions about how this code evolved. One concerns the code’s modern size, which must have passed through several intermediate and simpler stages. A plausible evolutionary progression is of a first ‘core’ coding for only four amino acids – each one signified by a base: guanine, cytosine, adenine or uracil (Higgs 2009). The 2nd letter of a triplet sequence appears most likely to have defined this first core (XNX; where N is an informational nucleotide and X are non-informational spacers: Massey 2006; Rodin and Rodin 2006; Wu et al. 2008). The triplet codon design reflects a physical constraint. Only mRNAs with 3-letter codons can physically accommodate the pairs of tRNA adaptors bringing together amino acid residues (Crick 1968; Travers 2006). Thus, the first code had only four ‘words’. Each one could have been spelled in 16 different ways, with only the second letter being the same and informational.

Higgs (2009) proposed that the doublet code first originated by adding G at the 1 st position: GAX = aspartic or glutamic acid, GCX = alanine, GGX = glycine, and GUX = valine. The remaining doublets then paired to other amino acids; and eventually converted to an all triplet code (Novozhilov and Koonin 2009; Sengupta and Higgs 2015; Supplement Fig. S1). A code that grows from 4 to 64 codons further implies that amino acids were likely added sequentially, from the first core to the 20th. To do so, the first and third letters of codon changed from being unused for informational purposes; to being used to specifically designate either an amino acid or a Stop signal. Assuming that the evolution for a singlet to doublet and then to a triplet was not instantaneous, means that there were mixed states. For the above first code, both GCX and any (non-G) XCX could code for alanine. The former codes as a doublet and the latter as singlet. This would mean that every triplet codon has always coded for something, but that early in code evolution the something was often the same thing.

The proposed orders in which amino acid entered the evolving code (Supplement Table S1) vary in being based on a consensus of potentially predictive factors (Trifonov 2000, 2004; Saier 2008), or inferences from amino acid frequencies in highly conserved proteins across the domains of life (Brooks et al. 2004; Liu et al. 2010; Zhao et al. 2020; Wehbi et al. 2024). However, the proposed entry orders neither answer why codons pair to particular amino acids, nor why the order would have occurred in a specific sequence.

The modern genetic code, nevertheless, does appear to reduce the overall probability that random mutations alter protein sequences. Indeed, Freeland and Hurst (1998) suggest that the modern code’s mutation patterns have a “one in a million” chance of having arisen through random pairing of codons to amino acids. Most, but not all, subsequent studies also propose adaptive scenarios for reducing mutation costs in the network of codon assignments (Zhu et al. 2003; Copley et al. 2005; Butler and Goldenfeld 2007; Stoltzfus and Yampolsky 2007; Novozhilov and Koonin 2009; Tlusty 2010; Kun and Radványi 2018; Fimmel et al. 2021; Radványi and Kun 2021; Freeland et al. 2023). Critical for adaptive scenarios is incorporating that mutations vary significantly in potential consequences (Crick 1966; Higgs 2009; Koonin and Novozhilov 2009). For example, a mutation of GCA to GCG still codes for valine, and thus likely has no effect on protein construction. In contrast, GCA to GUA exchanges valine for alanine (both hydrophobic), and GCA to GAA shifts to glutamic acid. Although both may affect protein structure, the latter substitution potentially has a larger effect on protein structure than the former. Additionally, the first is a transition across two pyrimidines, the second is a transverse where a pyrimidine replaces a purine. Because transitions occur more often than transverses (Freeland and Hurst 1998), mutational shifts to alanine are more expected than shifts to glutamic acid.

We suggest that within the code there are two mutations could be especially catastrophic for protein structure. One is to or from a Stop codon. Adding a Stop signal in the middle of a coding sequence would ‘break’ the produced protein. Removing the Stop could add a superfluous string of amino acids. Both might render the product useless for its evolved purpose. A second potentially catastrophic mutation is to or from the amino acid cysteine, which has the capacity for disulfide bonds (although these may have been less featured early in code evolution: Edwards et al. 2013). These often critically determine protein tertiary shapes essential for functioning. While adding or removing a cysteine may not be as dire in effect as with Stops, the results are still more likely to be consequential than shifts between any other pair of amino acids.

We accept that the genetic code has been evolutionarily selected to reduce the effects of mutations on protein synthesis. Overlooked, however, has been the selective advantage for assigning codons so as to especially reduce the number of mutational catastrophes. Thus, our “Catastrophic Mutation Minimization Hypothesis” (CMMH), is based on four propositions:

1. A Stop codon is an early feature of the genetic code, possibly already present at the transition from a singlet to a doublet code (Jimenez Sanchez 1995; Delarue 2007). Deviating from Higgs (2009), we propose adenine coded as a Stop signal rather than glutamic acid and evolved to occupy the UAX doublet (its current location). Thus, in a singlet code a transversion of C or U to A or a transition from G to A would produce a Stop signal. Catastrophic mutations could be relatively common in such a scenario, which could significantly negatively impact the overall viability of a code (Naora et al. 1987; Johnson et al. 2011). This could be a factor favoring the evolution of a more specific doublet code that not only adds more amino acids but also increases isolation of Stop signals from mutational consequences.
2. After cysteine enters the genetic code, a second catastrophic mutation becomes possible that affects the filling of still unpaired codons.
3. Amino acids enter only to codons that have unused letters. Explicitly differing from Trifonov (2004), we assume the evolution of the genetic code cannot readily replace or rearrange previously established amino acid/codon pairings (i.e., completely replace another amino acid at a doublet or triplet codon, but can share a doublet that is expanding to a triplet). To do so would often be too disruptive to protein synthesis across the entire genome (Crick 1968). Thus, if a pairing is established and used to make proteins, that relationship can become evolutionarily “frozen”.
4. For the purposes of this study, we test the CMMH against the most recent and comprehensive analysis of amino acid entry order for LUCA (Wehbi et al. 2024). Interestingly, Wehbi et al. (2024) also conclude that some codon to amino acid pairings suggest an origin in genetic codes that likely predate the LUCA code. For example, across all entry orders (Table S1), tryptophan (W) is the either the 19th or 20th amino acid added to the modern code. However, some ancient proteins whose syntheses are likely to predate LUCA are enriched in W (Wehbi et al. 2024). Thus, the entry of W into the modern code might reflect a late horizontal gene transfer from an older rival code that existed in a diverse ecological community varying in their codon/amino acid usage (Moody et al. 2024; Wehbi et al. 2024). As the LUCA code is the sole survivor, we can only test whether or not its entry order would generate competitive advantages through the CMMH. These could have been significant in favoring its universal adoption.

Given these four propositions, the CMMH makes a series of predictions as to the most likely codon to pair with a given amino acid in the construction of modern genetic code.

## CMMH Predictions and Results

### CMMH Prediction 1: Mutation Distance Significantly Correlates to Amino Acid Entry Order into the Genetic Code

The general prediction of the CMMH is that each sequential addition of an amino acid to the genetic code should be biased in pairing to codons that are the furthest mutational distance from a Stop codon. Using the latest and most comprehensive proposed order of addition (Wehbi et al. 2024), we correlate entry order to CMMH mutational distance scores (MDS) in modern genetic code. A triplet codon’s MDS to a Stop equals the number of mutations it would take to become a Stop (1 to 3) weighted by whether the mutation is a transition (more likely and given a value of one) or a transverse (less likely; value of two as in Freeland and Hurst 1998). Thus, the MDS of individual codons varies from 1 (one transition) to 6 (three transverses).

Note that these two measures are independent relative to each other. The Wehbi et al. amino acid entry order is derived independent of codon assignation, and a codon’s MDS is independent of which amino acid is paired to it. Thus, they are not covariates connected by an intrinsic significant relationship.

For comparison purposes, the Wehbi et al. (2024) rank order is also correlated against three earlier proposed entry rank orders that do not assign special roles to Stop and cysteine (Trifonov 2004; Saier 2008; Liu et al. 2010). Thus, the prediction here is that MDS produces a more significant fit with the Wehbi et al. rank order than do the previous orders.

### Results

**Fig. 1:**
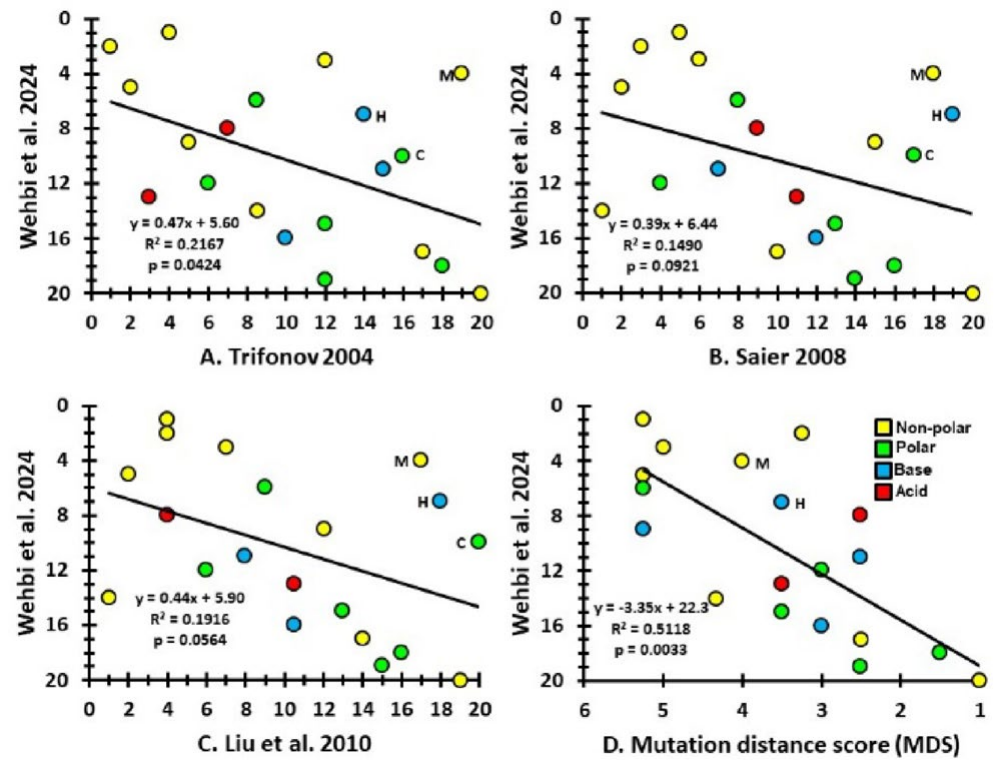
Entry order of 20 amino acids. Correlations to Wehbi et al. (2024), which is based on amino acid usage in highly conserved proteins across the domains of life (p values from Spearman Rank Correlation test). For all the earlier proposed orders (A-C), cysteine, histidine and methionine are predicted to enter much later than in Wehbi et al. Mutation distance scores (MDS) from the CMMH increase the further a codon is from either a Stop or cysteine codon. Because the CMMH predicts cysteine should be close to Stop, it is excluded from (D). Colors represent amino acid chemical properties

The MDS values across codons in the modern genetic code strongly correlates with the proposed rank order for amino acid entry in the genetic code of LUCA (Fig. 1D, *p* = 0.0033). Note that C is excluded because the CMMH predicts its entry is unique in that it should occur at the minimum MDS value.

In comparison, the correlations between previously proposed entry orders and Wehbi et al. (2024) are weaker to non-significant (Fig. 1A-C; *p* = 0.0424 to 0.0921). These differences between orders strongly reflect entry placements of two subsets of amino acids (Table S1). First, R, L and S are in a range of 1 st to 12th in earlier proposals, versus 12th to 16th in Wehbi et al. (2024). The earlier scenarios may have weighted them higher due to currently occupying the most codons (i.e., 6 for each). By the CMMH, these three fill the last five doublet codons. Second, all three earlier proposals have C, H and M as late additions to the genetic code (from 14th to 20th). This placement may follow from assumptions of non-availability through abiotic processes, thus requiring synthesis through complex metabolic pathways that may not yet exist. However, both C and M contain sulfur; which abiotic synthesis experiments generally did not make available (Wehbi et al. 2024). Moreover, H is important in metal binding and studies suggest basic cellular processes can synthesize it (Shen et al. 1990; Wehbi et al. 2024).

### CMMH Prediction 2: MDS Significantly Correlates to Both Early and Late Amino Acid Entry Order into the Genetic Code

Testing this prediction requires deriving likely antecedent doublet code networks, based on codon assignments in the modern network and the Wehbi et al. (2024) proposed entry order in LUCA (Table S1). In 9 of the 16 antecedent locations, the same amino acid is coded in the modern network by triplet codons sharing the first two letters, and with the third letter redundantly coding for the same amino acid across at least 3 of the 4 possible codons (Fig. S1). Thus, we project these nine codons in the antecedent first paired with the amino acid that occurs in the modern code. Wehbi et al. (2024) has cysteine as the 10th amino acid and its only current codons are UGC and UGU. Thus, we project UGX as being paired to cysteine. All amino acids added after cysteine as either doublet or triplet codons are predicted to favor an available codon that maximizes MDS from either cysteine, Stop, or both.

**Fig. 2:**
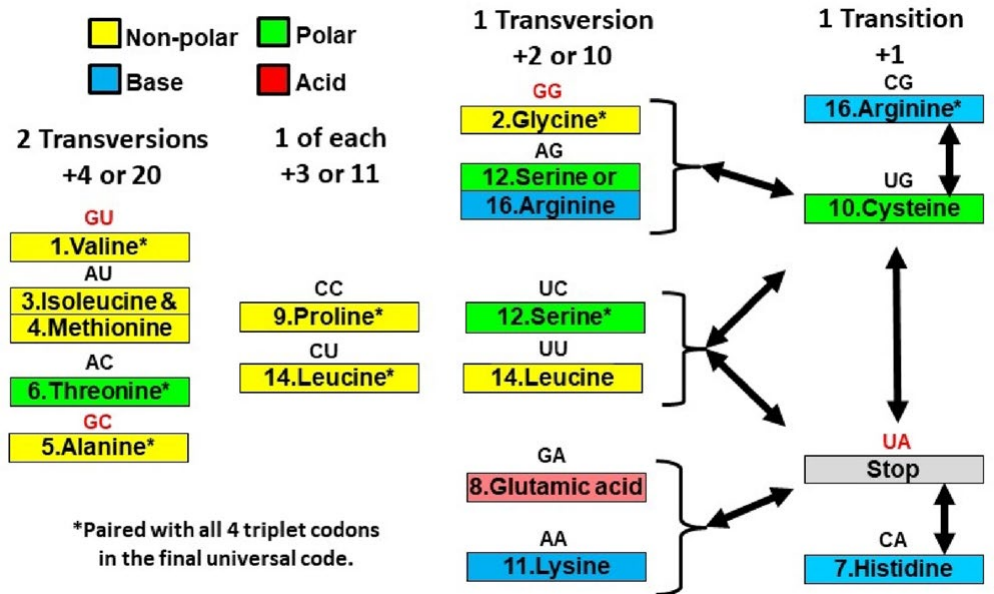
A proposed antecedent 16 codon, doublet genetic code. The numbers indicate the amino acid’s entry order from Wehbi et al. (2024). Red doublets indicate proposed members of the first singlet codon core (Higgs 2009). Mutation distance scores (MDS) are calculated relative to the Stop or cysteine codons. Transitions always add a one to the score; transversions add either a two or ten (see text for details). Serine or arginine are equally likely to have occupied the AG codon. Methionine’s apparent early entry makes it likely to have a triplet codon (AUG). Colors represent amino acid chemical property

Two of the three current Stop codons are UAG and UAA. UAC and UAU code for Y, the 18th amino acid added. Thus, we project UAX as being for Stop. For 4 doublets two amino acids are equally represented in the current code: E/D, H/Q, L/F and K/N. Here, the doublet codon is projected to the earlier one from Wehbi et al. (2024), and the other amino acid enters as a triplet codon. The remaining codon (AGX) is either R or S (Fig. S1). As both are already included in the antecedent code, alternative scenarios place them at AGX. Our proposed doublet code (Fig. 2) is identical to Copley et al. (2005), except they favored Q and N due to their simpler chemical structures.

### Results

**Fig. 3:**
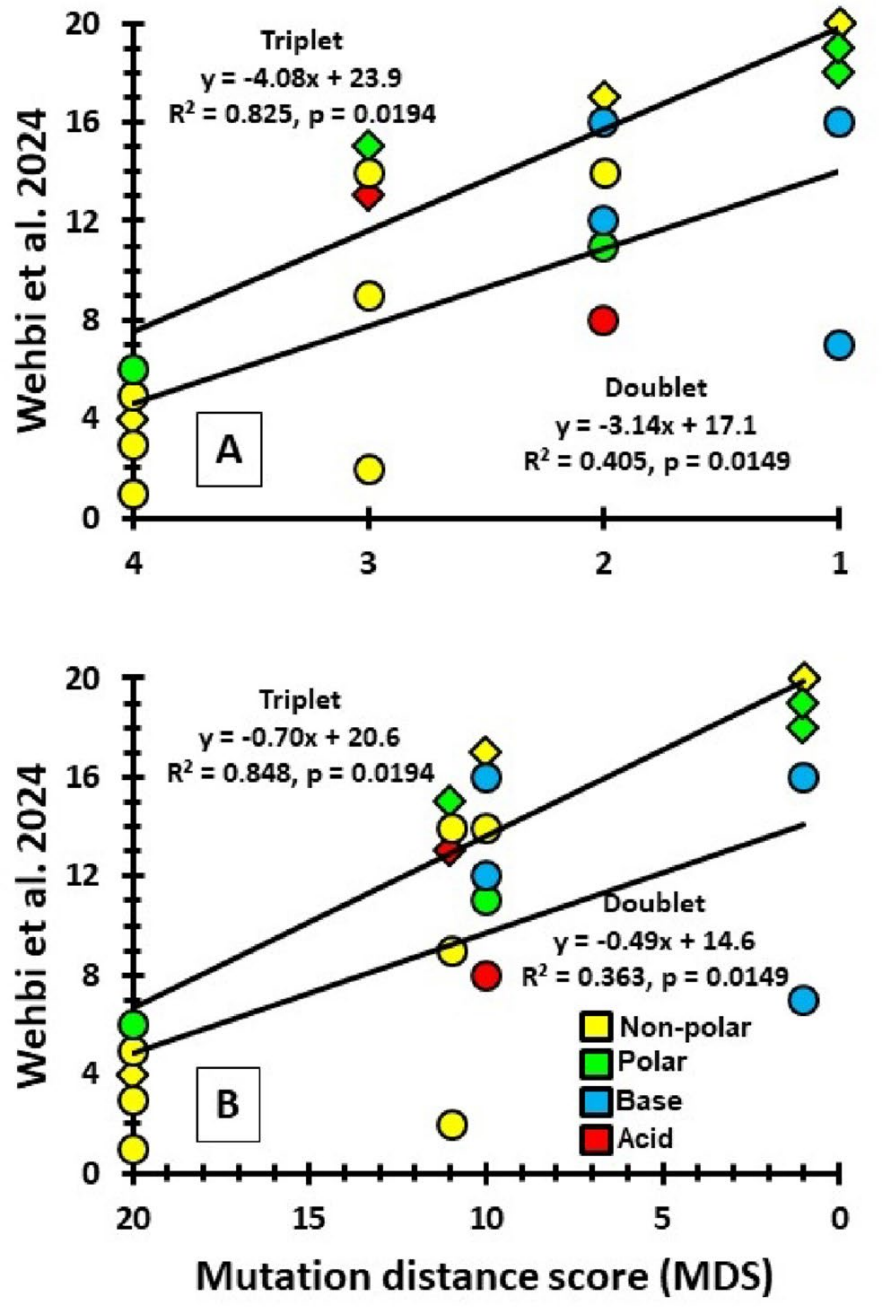
Amino acid entry order correlations for doublets (circles) and triplets (diamonds). Correlations are between mutation distance scores (MDS: Table S1) and the entry order (Wehbi et al. 2024). Circles are doublets, diamonds are triplets. The larger the MDS the further the codon is from either a Stop or cysteine codon. For the top panel (A), transverse mutations are given a value of 2; in the bottom panel (B) the value is 10. Shown with R at the AGX codon. With S at AGX, *p* = 0.0165 for doublets. (p values from Spearman Rank Correlation test) Colors represent amino acid chemical properties

The 20 amino acids can be divided into doublet (NNX) or triplet (NNN) groups, and MDS from the CMMH significantly correlates with both groups relative to their order of codon filling (Fig. 3). Predictions are robust as to whether transverse mutations are slightly or greatly less likely than transitions, and whether R or S is at the AGX codon.

### CMMH Prediction #3a: The Candidate 16-Codon Genetic Code Minimizes Network-Wide Mutation Costs

**Fig. 4:**
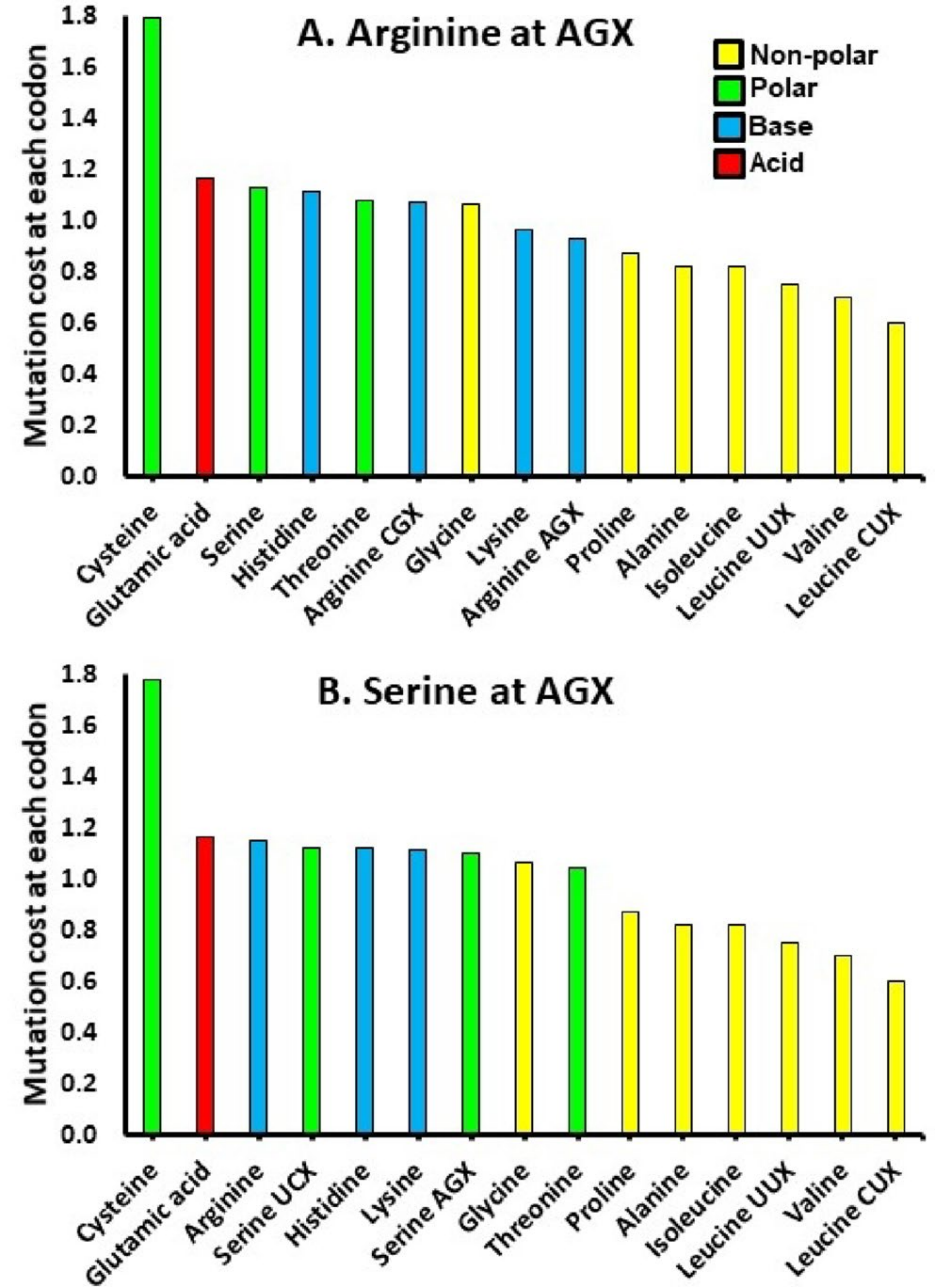
Per amino acid mutation costs in the proposed doublet genetic code. Values shown are for an amino acid paired to the proposed codon (from Fig. 2). Transitions are twice as likely as transversions. The CMMH proposes that network-wide mutation costs are minimized. Thus, individual amino acids could have lower mutation cost if located at other codons (e.g., cysteine, which is always the costliest amino acid in terms of mutations), but at a potential cost of increasing the costs of multiple other amino acids, and therefore, the overall network-wide cost. Colors define the chemical properties of the amino acid. The tendency of non-polar amino acids to occupy codons mutationally close to each other, clusters them far from the Stop codon and thus produces most of the lowest amino acid costs

For each doublet codon (except UAX Stop) we calculate the probability that in a given replication event the codon mutates to one of the 15 other possible doublets (this includes mutating to UAX). The cost for becoming a different codon depends on what the change produces. A mutation from CUX to UUX still codes for L, and therefore, the cost is zero. CUX to CCX is L to P, but as both are non-polar amino acids the cost is set to one. CUX to CAX is L to H, changing non-polar to basic, at an assigned cost of two. CUX to UGX or UAX is L to C or Stop. Any mutation to or from C is assigned a cost of three, as is any mutation to Stop. An exception to these last costs is that since C is polar amino acid, any mutation of C to or from another polar amino acid is less costly (i.e., 2.5), and that a mutation of C to Stop is the single most costly (i.e., a 4). Each codon adds to the network-wide mutation cost as the summed value of the 15 mutation probabilities multiplied by the above cost (Fig. 4; and see Supplement for details of the calculations).

We calculated the network-wide mutation cost of the above projected antecedent doublet code with either R or S at AGX. We also calculated scores if transverse mutations were 10x rather than 2x rarer than transitions. We assume that codons GCX, GGX, GUX and UAX represented a first core of A, G, V and Stop and 25,000 times randomly reassigned the remaining 12 codons to the other amino acids in the proposed antecedent and calculated the resulting network-wide mutation cost. The CMMH prediction being that the antecedent’s score should be less than a significant majority of the randomly generated values. See Supplement for simulation model details and the R code used.

### Results

The observed network-wide mutational costs more strongly support the CMMH for the proposed antecedent code when R is with codon AGX rather than S (Fig. S2 A-D). With R at AGX, 97.54% and 94.86% of randomly-generated codon assignments produce network-wide mutational costs greater than the value from the CMMH doublet network when transitions are respectively twice or ten times as likely as transverses. With S at AGX, the resultant percentages are 92.45% and 88.15%. As the CMMH makes a directional prediction, a one-tailed significance test is appropriate (i.e., more than 90%). Thus, three of the four scenarios for antecedent codes support the hypothesis that these proposed networks are significantly non-random relative to codon locations of Stop and cysteine. Figure S3 shows the best doublet networks for minimizing overall mutation costs.

### CMMH Prediction #3b

If there are no Stops and cysteine has the same costs as other polar amino acids, the antecedent doublet code will be more likely to be a result of random codon assignment. Therefore, we repeated the above simulations where mutations to or from cysteine have the same costs as for any other polar amino acid, and mutations to Stop are as costly as mutation to an amino acid in a different chemical group (i.e., 2). This parallels Freeland and Hurst’s (1998) analysis for the modern code that only incorporated a differential probability for transition versus transversion mutations.

### Results

If mutations to and from Stop and cysteine codons are assumed not to be catastrophic, minimizing network-wide mutational distances cannot as definitively reject a hypothesis of random pairing of codon to amino acid in the proposed antecedent doublet genetic code (Fig. S2 E-H). In only one scenario is the observed value less than 12% of the simulated values.

### CMMH Prediction #3c

The CMMH predicts placing cysteine at a codon closest to Stop will minimize network-wide mutation costs by creating a single negative pole within the codon network. Thus, across the random simulations from 3b, the CMMH predicts pairing cysteine with CAX or UGX (one transition removed from UAX), will result in lower network-wide costs than when paired to the other polar amino acids, threonine and serine. If cysteine is no different in cost to these other two polar amino acids, there should be no location bias that differs across the three. Note that we specifically made C to Stop mutations the costliest in the model to create a mathematically bias against placing C next to a Stop. Thus, if C is always less costly at a codon with a higher MDS, a positioning close to Stop must be due to reducing MDS at the other 14 codons.

### Results

**Fig. 5:**
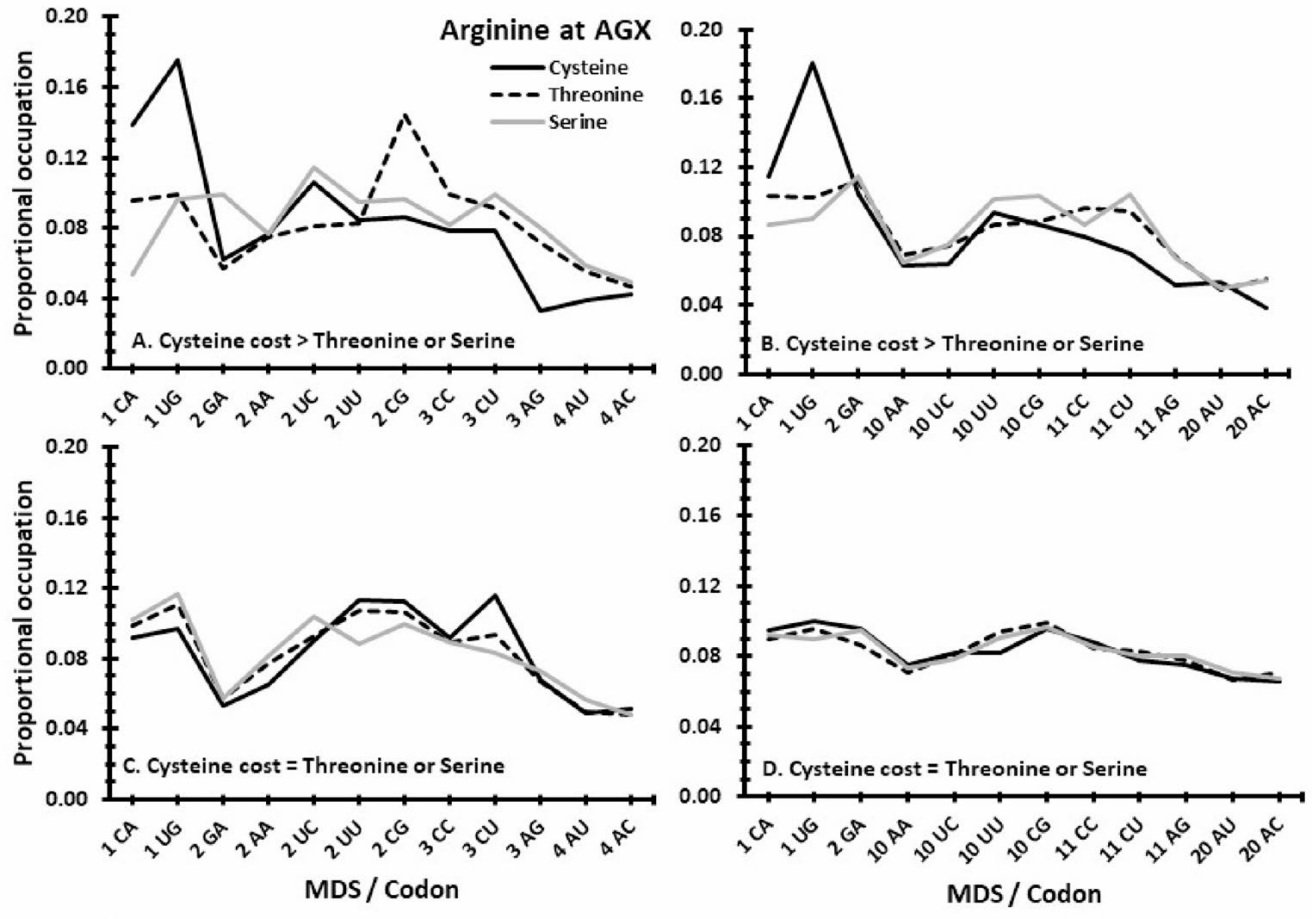
Predicting the codon placement of cysteine. In A & B, cysteine has a greater mutational cost than the other polar amino acids (threonine and serine). When randomly simulated with cysteine at UGX and arginine at AGX, 2.46% and 5.14% of codon arrangements have lower network-wide mutation costs than does a proposed antecedent code (Supplement Fig. 2A&B). In the subsets of those ‘better’ arrangements, cysteine is disproportionally more likely to be associated with either UGX or CAX (both are one transition mutation from UAX Stop), relative to the 10 other possible codon locations. Also, as the mutation distance to Stop increases across codons (ranked on the x-axis by mutation distance score), the probability of a cysteine being paired with that codon decreases. In contrast in C & D, cysteine is assumed to have the same costs as the two other polar amino acids. Random simulations under these conditions, predicts no preferential codon usage in the ‘better’ arrangements for any of the three polar amino acids. Cysteine is not predicted to be more likely associated with either UGX or CAX, and increasing codon MDS does not affect pairings to cysteine. X-axes rank codons by increasing MDS cost. In panels A and C, transverse mutations are more likely; in B and D, less likely

The simulations from prediction 3a produced codon networks in which their summed mutation costs are lower than the cost of the proposed antecedent doublet code (Fig. S2). They form sets of potentially ‘better’ options. In these sets, when cysteine has a greater mutational cost than other polar amino acids, it is more often found pairing with codons CAX and UGX (the only codons one transition from UAX) than threonine or serine (Fig. 5A, B; Fig. S4 A, B). When cysteine does not have a greater cost, then this bias is absent (Fig. 5C, D; Fig. S4 C, D). In summary, cysteine is found at a predicted codon consistent with its mutations imposing a relatively greater cost.

Intriguingly, if any amino acids were reassigned during the evolution of the modern code, cysteine may have been the most obvious candidate. Its adaptive function in forming disulfide bridges may have been rare or absent in determining protein structure at the point C was added to the genetic code (Edwards et al. 2013). Hence, mutations to or from C may not have been originally costlier than those involving any other polar amino acid. If so, the placement of C at UGX as the 10th added is not predicted by the CMMH (i.e., AGX would be a more parsimonious site, with UGX being the last codon to be used). Thus, an alternative scenario would be that C was first added to AGX, and then reassigned to the unused UGX as disulfide bridges became more common and important for protein shape – and replaced at AGX by the also polar serine.

### CMMH Prediction #3d

In the first genetic code only the second letter of a triplet is proposed to specify the amino acid (Higgs 2009). All mutations at the first and third letters have no effect because they are uninformative. We follow Higgs (2009) in presuming that the evolution of the doublet code begins with GNX, where N = C, G or U codes for alanine, glycine or valine respectively. We differ from Higgs in proposing the fourth informational doublet was UAX and Stop. This means that a mutation of GNX to GAX would still code for Stop (as the G in this pair would be uninformative). At this evolutionary point, coding for amino acids A, G or V would be predominantly how protein synthesis proceeds. Thus, the CMMH predicts that GAX would be an early codon in pairing with an added amino acid as the doublet code fills. With another amino acid at GAX, the original three become maximally mutationally isolated from a catastrophic Stop signal (i.e., GCX, GGX and GUX would require two mutations to become a Stop). However, for the GAX codon, any mutation at the first letter would produce a Stop signal: as UAX or XAX. To limit such outcomes, the CMMH also predicts that the codons AAX and CAX are early fills that complete the mutational isolation of the GNX row (Fig. S1).

Therefore, the CMMH predicts that as the doublet codons sequentially evolve pairings with new amino acids, the rows and columns (Fig. S1) would fill in relation to the UAX codon. The first row to would fill at GAX, and thereafter rows fill in order of mutational distance to UAX: row A as the first letter, followed by C and then U. For columns, the one where A is the second letter should be the first to fill. With six amino acids paired at these column and row positions, no XAX codon or codon without an A at the second letter could code for a Stop. Functionally, the evolving doublet genetic code could have had only a single Stop codon well before it was completely filled. Only one Stop would minimize network-wide mutation costs.

### Results

Both CMMH predictions are supported (Table 1). Rows fill in the predicted order from the first letter of G, to the last row of U. Also, the column with the second letter A fills in earlier than columns C, G or U.

**Table 1:**
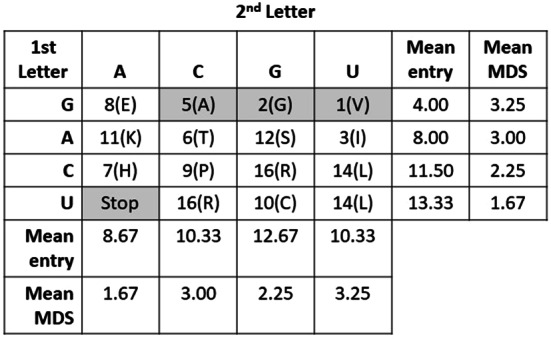
CMMH predictions for the proposed 16-codon genetic code by row and column

Entry order of the amino acids (in parentheses) as proposed by Wehbi et al. (2024). The CMMH predicts mean entry order for rows increases as mean mutation distance (MDS: with scoring transitions as 2x more likely to happen than transversions) to Stop (UAX) decreases. The CMMH also predicts that the mean entry order for the A column will be earlier and at a closer MDS than columns C, G and U to preclude any XAX codons from producing a Stop signal. Means are calculated from the unshaded cells; shaded cells are the proposed elements of the first genetic code

### CMMH Prediction #4: As Amino Acids are Added to the Antecedent Code, they Pair with the Available Codon that has the Greatest MDS

For the first 14 codon locations that were filled in the proposed antecedent code (Fig. 1), we computed the mean MDS of the still available codons; from 15 when V is added to 2 when R is added. This mean is compared to the MDS values from the observed codon/amino acid pairing. If MDS had a positive effect on the pairings, then the observed values should be consistently greater than the mean score. If MDS had no significant effect, then the observed should not be significantly greater than the mean. I.e., the observed outcome should be equally likely to be above or below the mean score.

### Results

**Fig. 6:**
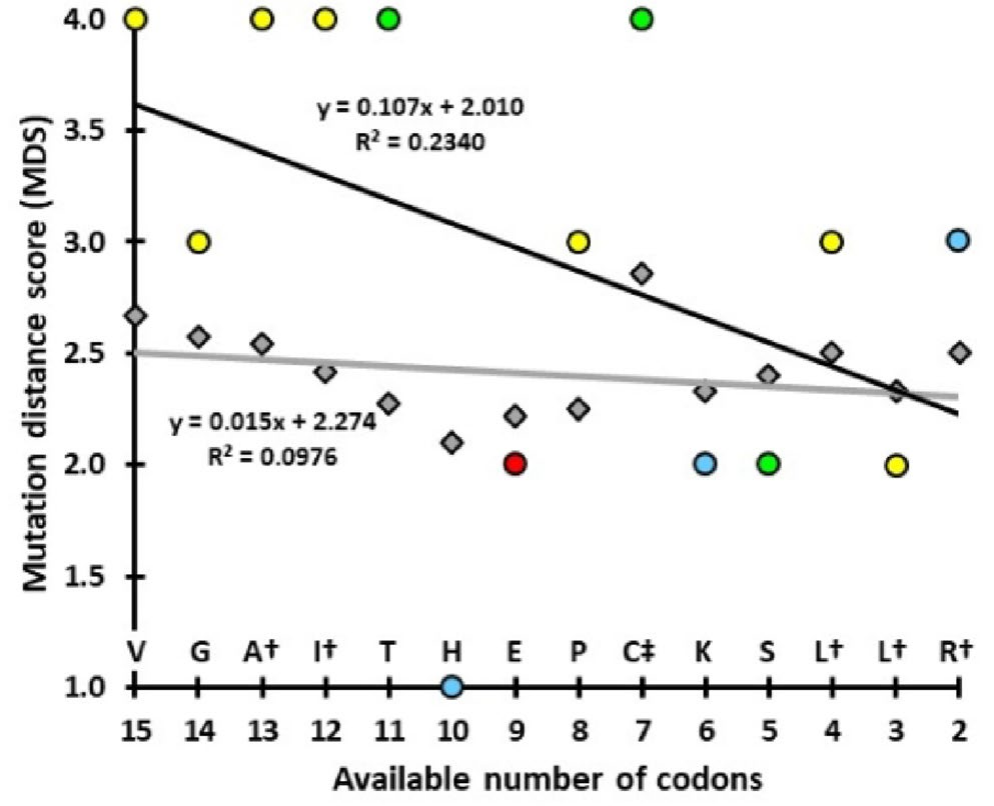
Codon selection by sequentially accumulating amino acids in the proposed doublet code. The grey diamonds represent the mean MDS across the available codons for pairing to an amino acid. The colored circles are the MDS of the codon for the predicted pairing from Fig. 2 (yellow = non-polar, green = polar, blue = base, and red = acid). Trend lines are given for each. Overall, the observed MDS values are significantly greater than the mean values: Wilcoxon Signed-rank test, *p* = 0.0354. This analysis is with R at the AGX codon. ^†^indicates the amino acid is only one transition mutation removed from an admitted amino acid in the same chemical group. ^‡^Cysteine is predicted by the CMMH and has a codon (UGX) that is one transition mutation distant from Stop and, therefore, realizes its ‘best’ mutation score

The CMMH predicts that when an amino acid enters the genetic code, it should be paired with an available codon with the largest MDS (except for C, where the opposite is predicted). Across the first 14 additions to the proposed antecedent this prediction is significantly supported (Fig. 6). Amino acids consistently pair with a codon that has an MDS value larger than the mean MDS of all available codons. This is particularly evident in the first five additions. The sixth (histidine) is the major outlier to this prediction. (Note that if H is excluded, the significance level of the Wilcoxon Signed-rank test increases to *p* = 0.0130).

### CMMH Prediction #5: Expansion from a Doublet to a Triplet Code is Affected by Stop Codons

Redundancy in amino acid coding at the 3rd letter differentially occurs relative to entry order, such that mutationally safer triplet codons are assigned before triplets close to Stop or C. The last amino acids to enter the genetic code will be assigned to triplet codons that are mutationally closest to a Stop or cysteine codon. Overall, network-wide mutation costs are minimized if these amino acids are also the least used to make proteins.

### Results

The mean MDS to Stops and C’s in the modern code of the 48 triplet codons of the 13 amino acids placed in the antecedent code is 4.12 ± 0.93 and 17.72 ± 6.12 for likely and unlikely transverse scenarios. For the 11 codons of the six amino acids added later, the means are: 3.07 ± 0.92 and 11.80 ± 5.54. The differences between early and late additions are significant by t-tests (t_57_ = 3.386, *p* = 0.0013 and t_57_ = 2.940, *p* = 0.0047 for the two transverse scenarios). Thus as predicted by the CMMH, the last amino acids added to the code are significantly closer by mutation to Stop and C than the amino acids in the first core and the doublet ancestral code.

The overall occurrence of catastrophic mutations would be reduced if codons in the one-transition-away slots from the C or Stop codons are rarely used. In support of this CMMH prediction, C, H and R occupy the closest positions in the 16-codon structure (Fig. 2), and these are also the least used amino acids in Archaea, Bacteria, Eukaryotes and Viruses (Morimoto and Pietras 2024). Similarly, W is the consensus 20th amino acid and among the least used in protein synthesis (Koonin 2017). Its codon, UGG is one transition from Stop UGA and UAG.

### CMMH Prediction #6: Usage of Triplet Codons Close to Stop and Cysteine

Reducing the possibility of catastrophic mutations could affect: (a) Which doublet codons evolve to be redundant for the same amino acid at their 3rd letter; (b) Which amino acids pair to triplet codons that are mutationally close to Stop codons; (c) Favoritism towards using pyrimidines (C and U) at the 3rd letter to code for late entry amino acids; and (d) Which codons are assigned to amino acids added after the 20th entry.

### Results

Stop codons appear influential on four general patterns found in the modern triplet codon network.

- Nine of the 16 NNX doublets evolve into triplets where at least three of the 3rd letters produce redundant codons for the same amino acid (Fig. 2). Across their 36 triplet codons, 12 are three mutations removed from any Stop codon, 20 are two mutations removed, and 4 are separated by one mutation (and only one is a transition). Ergo, this triplet codon region filled in with a minimal added risk for catastrophic mutations to a Stop signal. The mean MDS is 2.222 ± 0.423 (Table S2). For the other seven NNX doublets, no amino acid is associated with more than two codons in the triplet code. Across the 25 non-Stop triplet codons, none are three mutations removed from any Stop codon, 11 are two mutations removed, and 14 are separated by one mutation. Their mean MDS is 1.393 ± 0.283. The difference in mean MDS’s across the two groups is statistically significant (Table S2, unpaired t-test: t_14_ = 4.452, *p* = 0.0005). An alternative hypothesis is that the first codons to fill in the doublet code were also the ones to become redundant at the 3rd letter (Table S2). Although there is a negative trend for entry order to correlate with triplet codon redundancy, it is not statistically significant (MDS = −0.048 (entry order) + 2.328; F_1,13_ = 2.981, *p* = 0.1079). Earlier-filled codons first associated with H, E, C and K do not become redundant, while later-filled codons associated with L, S and R do so. Thus, MDS is a significantly better predictor of expansion patterns in the triplet code than is the order in which codons filled.
- From the perspective of the three Stop codons, nine triplets are one transition away. Four are alternative Stop codons; two are for Q; two are for W; and one is for R. Comparatively, in a genetic code with only one Stop codon, all three transitions would be to an amino acid. Thus. a three-fold increase in Stop codons less than doubles the number of the most common mutations that could cause a catastrophe. For the two C codons, six triplets are one transition away. Two are alternative C codons; 2 are for Y; and 2 are for R. Amino acids Y, Q and W are the last three added (Wehbi et al. 2024), and CMMH accurately predicts they should have the worst slots by mutation distance (Table 2). Similarly, R as the last to enter a doublet code predictably fills the only available codon. When R expands to a triplet code, three codons (AGA, AGG and CGG) are more than one transition from either C or Stop. Intriguingly, an extensive human genome survey finds those three arginine codons are about equally common: totaling 62.71% of all codons (Schulze et al. 2020). Two codons one transition from C (CGC and CGU) comprised 26.34%, with the one codon close to Stop (CGA) at10.94%. It would be interesting to see how common across species is such a R codon usage bias.
- In the modern genetic code, the three triplet codons of the Stop signal all have A or G as the 3rd codon letter. If the current usage pattern also prevailed when the last six amino acids were added, then the sequential pairing of D, N, F and Y to codons with C or U at the 3rd letter, maximally increases their mutational distance from the Stop codons (Table 2). Only the last two amino acids added (Q and W) use G and A as the 3rd letter.
- The CMMH assumes and requires no ‘capture’ of codon sites throughout the evolution of the genetic code: I.e., breaking an existing amino acid/codon connection and replacing it entirely with another. The CMMH progresses by converting unused letters in a codon to being informational. In contrast, the entry orders of earlier proposals explicitly require multiple capture and codon reassignment for late-addition amino acids (Trifonov 2004). Such codon reassignments do (rarely) occur. The observed shifts, however, support CMMH. Stop shifts to amino acids have been noted 27 times, compared to 7 shifts between two amino acids, and two shifts from amino acid to Stop (Sengupta and Higgs 2015). Also, the rare code additions of a 21st (Selenocysteine) and 22nd (Pyrrolysine) amino acid occurred at Stop codons UGA and UAG, respectively (Sengupta and Higgs 2015). Evolutionarily, filling Stop codons can generate dual benefits – adding intrinsic amino acid properties for protein construction and reducing catastrophic mutation risk.

The results across all the predictions support the initial assumption that antecedents to the modern genetic code had specific Stop signal(s). Indeed, if future evidence conclusively shows that Stop codons are post-LUCA then the pre-LUCA patterns reported here will need an alternative explanation.

**Table 2:** CMMH predictions for amino acid and codon pairs

Codons (initially assigned as doublets)	Amino acids	Relationship to CMMH prediction regarding doublet Stop or cysteine codons
GU* GG* GC*	Valine (V) Glycine (G) Alanine (A)	All 3 consistent. All three pair with codons that have an MDS greater than mean of all available codons (Fig. 6) and create a nonpolar neighborhood.
AUA, AUC, AUU	Isoleucine (I)	Consistent. Codon has maximum available MDS from UAX (Fig. 6), and is in a nonpolar neighborhood.
AC*	Threonine (T)	Mixed. Codon has maximum available MDS from UAX (Fig. 6), Although initial placement is optimal, it increases network-wide MDS when the code fills.
CAC, CAU	Histidine (H)	Not consistent. Codon located one transition from UAX with multiple better locations available at entry.
GAG, GAA	Glutamic acid (E)	Not consistent. Better codons (CCX or CUX) were available at entry.
CC*	Proline (P)	Consistent. Codon in nonpolar neighborhood and has an MDS greater than mean of all available codons (Fig. 6).
UGC, UGU	Cysteine (C)	Consistent. Minimal MDS to UAX, and adding the most to the network-wide mutation scores (Fig. 4).
AAA, AAG	Lysine (K)	Not consistent. Codon has an MDS less than the mean of all available codons (Fig. 6).
UC*, AGC, AGU	Serine (S)	Not consistent. Codon has an MDS less than the mean of all available codons (Fig. 6). If S also fills AGX, that codon is 2 transverse mutations distant from Serine’s other codon, UCX.
CU*, UUA, UUG	Leucine (L)	Partly consistent. Codons in nonpolar neighborhood and one has an MDS greater than mean of all available codons (Fig. 6).
CG*, AGA, AGG	Arginine (R)	Consistent. Either filling the last or last two available codons in the doublet code.

Codons (assigned only as triplets)	Amino acids	Relationship to CMMH prediction regarding triplet Stop codons UAA, UAG and UGA
AUG	Methionine (M)	Consistent. As the first triplet codon, it is in a shared nonpolar neighborhood and its Start function is distant from Stop triplets.
GAC, GAU	Aspartic acid (D)	Consistent. Codons are at maximum available MDS (2 transverses to Stop triplets), and creates an acid neighborhood with E.
AAC, AAU	Asparagine (N)	Consistent. Codons are at maximum available MDS (2 transverses to Stop triplets).
UUC, UUU	Phenylalanine (F)	Consistent. Codons are at maximum available MDS (2 transverses to Stop triplets).
UAC, UAU	Tyrosine (Y)	Consistent. Codons are one transversion from Stop triplets rather than one transition.
CAA, CAG UGG	Glutamine (Q) Tryptophan (W)	Consistent. Only available codons are one transition from Stop triplets.

*Indicates that in a triplet codon all the 3rd letters code for the same amino acid

The amino acid entry order is: V, G and A together as the first core code (Higgs 2009); the other doublets from Fig. 2; and all the triplets in order from Wehbi et al. (2024). In the triplet code, 52 of 64 codons align to the 13 amino acids and Stop signal present in the antecedent code. The CMMH predicts that entering amino acids should favor codons with the maximum distance score (MDS) from UAX (Stop) or UGX (cysteine) among the available unpaired codons; and clustering codons by affinity as mutations to amino acids of the same chemical type are less costly than across types. A fourth prediction is specific to cysteine. It should locate one transition mutation removed from Stop and add the most cost to the network-wide score (Fig. 4)

## Discussion

Multiple lines of evidence are consistent with a proposition that features associated with the codons UAX and UGX had disproportionate effects on the codon to amino acid pairings that created the modern genetic code. The Catastrophic Mutation Minimization Hypothesis posits that a Stop signal at UAX and the amino acid cysteine at UGX are the features in question. Both are vital and necessary component parts for building biologically useful proteins. Random mutations to and from both, however, can also cause catastrophic failure for protein synthesis. Thus, the CMMH predicts that this risk favors the semi-isolation of their codons by mutation distance score (MDS) from the majority of amino acids. A relatively early addition for both, would have added a negative pole in the genetic code. This creates a selective advantage for favoring particular codons for subsequent amino additions to either maximize their MDS or minimize network-wide mutation costs. In total, the codon to amino acid pairings are consistent across the breadth of CMMH predictions for 16 of the 20 amino acids (Table 2). To date, no other alternative hypothesis has reached this level of specific prediction. Note that the CMMH predicts pairings based on minimizing costs, which are calculated as mutational distances to Stop, cysteine, and other amino acids with like or unlike chemical properties. The CMMH as formulated here does not necessarily predict within a chemical grouping an entry order (e.g., why T, P, and E would have been predicted to be added to the code at ACX, CCX and GAX instead of S, L and D). Other factors such as stereochemical affinities may better predict amino acid identity (Harrison et al. 2022; Halpern et al. 2023).

The CMMH and Wehbi et al.’s (2024) amino acid entry order into LUCA are mutually reinforcing through using different methodologies and objectives. Theirs discern an order based on the premise that amino acid prevalence in highly conserved (and presumably ancient) protein sequences mirrors their entrance into the genetic code of LUCA (Wehbi et al. 2024). We test whether the codon locations of those entries follow from well-observed facts that mutations vary in likelihood and potential impact on protein structure. The CMMH is supported in terms of historical congruence, and it provides a simple and powerful evolutionary explanation for how those entries sequentially structured codon networks.

### Stop Codons Present in Pre-LUCA Genetic Codes

To our knowledge, there is neither definitive evidence for a Stop codon being present in genetic codes simpler than the modern 64-codon version, nor for a late post-LUCA addition of Stops. There are circumstantial arguments for each position. Stop codons are only plausible in the presence of an translational mechanism, which may have arisen only with LUCA (Higgs 2009). Also, Stop codons require specific releasing factors that terminate protein construction (Nakamura and Ito 1998; Zhouravleva and Bondarev 2013). These factors differ in bacteria versus archaea/eukaryotes to the extent that they are unlikely to have evolved from the same ancestral protein (Inagaki and Doolittle 2000). Their origin, therefore, suggests a separate and post-LUCA evolution of Stop codons after the addition of all 20 amino acids. However, it is also plausible that translation-similar mechanisms first arose in RNA world (Wolf and Koonin 2007; Fer et al. 2025) or a nucleopeptide world (Douglas et al. 2025). If so, these could have been integrated into even the earliest genetic codes. Also, it is possible the releasing factors evolved separately post-LUCA to replace an earlier, shared system – possibly in the form of a modified tRNA-like molecule (Burroughs and Aravind 2019). Additionally, the fact that the three Stop codons are universal across all organisms would be more consistent with a pre-LUCA origination (Burroughs and Aravind 2019). Finally, if Stop codons were absent from pre-LUCA codes, then what other feature of the UAX codon location could have produced the consistency in effects *as if* a Stop were present?

### Frozen Accidents, Abiotic Synthesis and Stereochemistry

Several features of the genetic code suggest that alternative or complimentary factors are present in addition the CMMH. For example, H, E, K and S are at codons when, at their entry, multiple other codons were available that were better in terms of MDS or minimizing network-wide mutation costs. Their codon locations are better explained as being frozen accidents (Crick 1968). Stochasticity may have also determined the first codon locations and subsequent amino acid associations. If three core amino acids all have G as their 1 st codon letter then UAX as the Stop doublet codon is as predicted by the CMMH based on mutational distance. However, if by chance the first core amino acids had been coded into UGX, UCX and UUX, the Stop codon might have been GAX and the entire resulting codon network could be inverted from that present today.

The earliest organisms may well not have had the enzymatic ability to make their own amino acids. Hence, they would need to rely on what was available through abiotic processes. At least 10 of the 20 amino acids could have been environmentally available, and these 10 also have the lowest free energy (Koonin and Novozhilov 2009, 2017). Nine are in the proposed ancestral code (Fig. 2) In another pattern, hydrophobic amino acids are over-represented (All 3 in the core code, 7 of the 14 in the doublet code, and 9 of the final 20). The CMMH makes no prediction about frequencies of amino acid chemical groups. Nevertheless, the hydrophobic bias suggests their selective importance and possible exchangeability for protein structure. They also cluster together in the code more so than do the multiple polars and bases (Fig. 2).

The stereochemical hypothesis (SH) proposes that the physical properties of mRNA, tRNA and amino acids are predictive of optimal codon use (Yarus et al. 2009; Halpern et al. 2023). Several relationships are consistent with the SH. The evolution of redundancy at 3rd letter would require a minimal change in tRNA’s that are already transporting the ‘correct’ amino acid. Glutamic and aspartic are both acids and share GAX codons. Similarly, the least stable DNA codon for effective base-pairing is UAX, and therefore, would be the most obvious candidate for Stop codons which would need no matching tRNAs (Travers 2006). However, at best the SH can match only 5–6 amino acids to a particular codon (Koonin 2017; Di Giulio 2022). Furthermore, in examining the entirety of modern code (Di Giulio 2022; Di Giulio 2024) finds more codon to amino acid pairings that are contradictory to the SH rather than being supportive.

### Antecedent Code Capabilities and Features

Although a doublet code is presented as a definitive stage in the evolution of the modern code, it might never have existed in such a pure form. Clearly, an early entry of M as the 4th amino acid (Wehbi et al. 2024) suggests that the AUX codon was already functioning as a triplet and perhaps also with a Start function. Similarly, the SH would predict an early expansion of GAX into a triplet to accommodate E and D, as the 8th and 13th amino acids in the code (Table S1). Finally, the early amino acids at greatest MDS from Stop may well have also evolved into redundant triplets while some NNX codons remained unused. This would have precluded adding amino acids 15–20 at those distant codons. Thus, an ancestral genetic code may never have existed as only 16 doublet codons, and instead was always evolving as a mixture of doublet and triplet codons.

Nevertheless, LUCA with only a subset of the modern code’s amino acids could still potentially express the majority of the characteristics present in the modern code. To the degree that Fig. 2 accurately reflects a transitional form of the genetic code, it would have:

- Start and Stop codons, both present and separated by maximal mutation distance.
- Representative amino acids with all four chemical properties: hydrophobic, hydrophilic, acidic and basic.
- Ability to form disulfide bonds – important for creating tertiary structure in proteins.
- Histidine as the first amino acid capable in binding multiple metals to proteins.
- An early presence of Methionine in the code, which could reflect a primordial environment rich in H_2_S; facilitation of S-adenosylmethionine (SAM) biosynthesis and SAM usage (Moody et al. 2024; Wehbi et al. 2024); suitability of the AUG codon for a Start location (Demongeot and Seligmann 2020); and as the donor to methylate other amino acids (primarily arginine and lysine; which are also both present). Thus, LUCA and pre-LUCA organisms could theoretically express epigenetic gene regulation (which may have arisen in RNA world, as mRNAs exhibit epigenetic modification: Garber 2019).
- Stop and cysteine in close proximity limits catastrophic mutations at the genome level.
- No need for future codon capture and amino acid replacement; events that are likely to be highly disruptive for protein synthesis (Crick 1968).

In summary, the CMMH supports an adaptive co-evolutionarily model for the genetic code. Amino acids enter into the code based on their useful properties for making complex protein products (Di Giulio 2022; Di Giulio 2024), with chemical availability and fit being a constraint or useful added benefit. Simultaneously, they are paired to codons to reduce negative mutation consequences – and especially those mutations that produce the most profound negative outcomes. Overall, a simpler antecedent code would have broadly compared to the modern code in its features and capabilities. These evolutionary events would have occurred in the pre-LUCA world, and thus possibly more than 4.5 billion years ago (Mahendrarajah et al. 2023). The expansion to LUCA’s universal code with 64 codons for 20 amino acids was, therefore, likely an evolutionary fine tuning of an already high-functioning system rather than a major increase in the capacity and ability to create adaptive proteins.

## Supplementary Information

Below is the link to the electronic supplementary material.

Supplementary Material 1
