# Supplementary material for: Avoiding Catastrophic Mutations Accurately Predicts Amino Acid to Codon Pairing
Source: J Mol Evol. 2025 Dec 20;94(1):164–76. doi: 10.1007/s00239-025-10294-0 (PMC12920281; doi:10.1007/s00239-025-10294-0)
Supplement: Supplementary file 1 — Supplementary Material 1 [file 239_2025_10294_MOESM1_ESM.docx]

**Supplement file.**

**Previous proposed entry orders of amino acids to the genetic code.**

Wehbi et al. (2024) is considered as the most authoritative (see text for details) and other proposed orders are correlated against theirs (Fig. 3).

**Supplement Table S1.** **Proposed amino acid entry orders**. Amino acids become part of the genetic code as either doublets (the antecedent code) or as triplets (for the modern code). Mutation distance scores (MDS) are an increasing positive value relative to the location of Stop or cysteine codons (see text for details). The values for triplet distances are the means of the least mutation distances to either Stop or cysteine (C).

| Entry Order | Trifonov (2004) | Saier (2008) | Liu et al.^†^ (2010) | Webli et al. (2024) | Codon size | Doublet MDS | Triplet MDS | Codon # |
| --- | --- | --- | --- | --- | --- | --- | --- | --- |
| 1 | G | L | L | V (Valine) | Doublet | 4 | 5.25 | 4 |
| 2 | A | A | A | G (Glycine) | Doublet | 3 | 3.25 | 4 |
| 3 | D | G | G* | I (Isoleucine) | Doublet | 4 | 5 | 3 |
| 4 | V | S | V* | M (Methionine) | Triplet |  | 4 | 1 |
| 5 | P | V | E* | A (Alanine) | Doublet | 4 | 5.25 | 4 |
| 6 | S | I | S | T (Threonine) | Doublet | 4 | 5.25 | 4 |
| 7 | E | K | I | H (Histidine) | Doublet | 1 | 2.5 | 2 |
| 8 | L* | T | K | E (Glutamic acid) | Doublet | 2 | 2.5 | 2 |
| 9 | T* | E | T | P (Proline) | Doublet | 3 | 4.25 | 4 |
| 10 | R | F | D** | C (Cysteine) | Doublet |  |  | 2 |
| 11 | N** | D | R** | K (Lysine) | Doublet | 2 | 2.5 | 2 |
| 12 | Q** | R | P | S (Serine)^††^ | Doublet | 2 & 2 | 2 & 3 | 4 & 2 |
| 13 | I** | N | N | D (Aspartic acid) | Triplet |  | 3.5 | 2 |
| 14 | H | Q | F | L (Leucine)^††^ | Doublet | 2 & 3 | 2.5 & 4.25 | 2 & 4 |
| 15 | K | P | Q | N (Asparagine) | Triplet |  | 3.5 | 2 |
| 16 | C | Y | Y | R (Arginine)^††^ | Doublet | 1 & 2 | 2.25 & 2.5 | 4 & 2 |
| 17 | F | C | M | F (Phenylalanine) | Triplet |  | 2.5 | 2 |
| 18 | Y | M | H | Y (Tyrosine) | Triplet |  | 1.5 | 2 |
| 19 | M | H | W | Q (Glutamine) | Triplet |  | 1.5 | 2 |
| 20 | W | W | C | W (Tryptophan) | Triplet |  | 1 | 1 |

* and ** are considered as ties by the authors.

^†^ Order updated in Zhao et al. (2020) as groups of amino acids, but were not presented in a 1-20 series. The only clear difference is C is placed after I, F and Y and before M and H, but it is not stated as to where their groups enter relative to the other amino acids.

^† †^L always occupies 2 doublets. R and S each have a unique doublet and one or the other occupies AG as a second codon.

**Supplement Table S2.** Number of mutations needed to go to a Stop triplet codon. Amino acid proposed as occupying the antecedent doublet code (if more than one given, the first is the original pair to the doublet). For the third letters in a codon, the coded for amino acid is given with the minimum number of mutations (in parentheses) to a Stop triplet codon.

| **Amino** | **Entry** | **Doublet** | **3^rd^ letter:** |  |  |  |  |
| --- | --- | --- | --- | --- | --- | --- | --- |
| **Acid(s)** | **order** | **Codon** | **G** | **C** | **A** | **U** | **Mean** |
| V | 1 | GU | V (2) | V (3) | V (2) | V (3) | 2.5 |
| G | 2 | GG | G (2) | G (2) | G (1) | G (2) | 1.75 |
| A | 3 | GC | A (2) | A (3) | A (2) | A (3) | 2.5 |
| I | 4 | AU | M (2) | I (3) | I (2) | I (3) | 2.5 |
| T | 5 | AC | T (2) | T (3) | T (2) | T (3) | 2.5 |
| H/Q | 6 | CA | Q (1) | H (2) | Q (1) | H (2) | 1.5 |
| E/D | 7 | GA | E (1) | D (2) | E (1) | D (2) | 1.5 |
| P | 8 | CC | P (2) | P (3) | P (2) | P (3) | 2.5 |
| C/W/Stop | 9 | UG | W (1) | C (1) | Stop | C (1) | 1.0 |
| K/N | 10 | AA | K (1) | N (2) | K (1) | N (2) | 1.5 |
| S | 11 | UC | S (1) | S (2) | S (1) | S (2) | 1.5 |
| L | 12 | CU | L (2) | L (3) | L (2) | L (3) | 2.5 |
| L/F | 13 | UU | L (1) | F (2) | L (1) | F (2) | 1.5 |
| R/S | 14 | AG | R (2) | S (2) | R (1) | S (2) | 1.75 |
| R | 15 | CG | R (2) | R (2) | R (1) | R (2) | 1.75 |
| Stop/Y |  | UA | Stop | Y(1) | Stop | Y(1) | 1.0 |

**The simulation model for the antecedent genetic code**.

*A. Calculating mutation probabilities.*

1. Codons are NNX with a 1/3^rd^ chance of mutating each of the three positions. Mutations at the X position have no effects.

2. Calculate probability of one or two functional mutations across NNX.

3. If one mutation, calculate probability of a transition or transverse. In the ‘likely’ scenario, transitions are 2x as common as transversions; in the ‘unlikely’ scenario, they are 10x as common.

4. If two mutations, calculate the probabilities for one of each, two transitions, and two transverses.

*B. Codon location probabilities.*

For every codon position, the distribution of the other 15 locations are as follows (Note that the codons for amino acids G, A, V and Stop are fixed across simulation runs):

- Two are one transition away (mutations equally likely across codons: 50% for each one).
- Four are one transverse away (25% chance for each codon).
- Four are one of each away (25% chance for each codon).
- One is two transitions away (100% chance).
- Four are two transverse away (25% chance for each codon).

The probability that a given codon will mutate to any of the other 15 codons is given in Table S2.

**Table S2: Codon mutation probabilities**. Transitions are assumed to be either twice or ten times as likely as transversions. The probabilities of a given mutated codon resulting is a function of the likelihoods of 1 or 2 mutations occurring at a codon, whether each mutation is a transition or a transverse, the mutational distance between the mutating and resulting codon, and the number of codons located at that distance (i.e., Likelihood x Proportion in the table).

|  | **Likelihood** | |  |  | **Mutated codon** | |
| --- | --- | --- | --- | --- | --- | --- |
| **Mutational distance** | **2x** | **10x** | **Codons** | **Proportion** | **2x** | **10x** |
| 1 Transition | 0.29630 | 0.40404 | 2 | 0.5 | 0.14815 | 0.20202 |
| 1 Transverse | 0.14815 | 0.04040 | 4 | 0.25 | 0.03704 | 0.01010 |
| 1 each | 0.04938 | 0.01837 | 4 | 0.25 | 0.01235 | 0.00459 |
| 2 Transitions | 0.04938 | 0.09183 | 1 | 1 | 0.04938 | 0.09183 |
| 2 Transversions | 0.01235 | 0.00092 | 4 | 0.25 | 0.00309 | 0.00023 |
| Total | 0.55556 | 0.55556 |  |  |  |  |

*C. Mutation cost*.

1. Mutations to the same amino acid have zero cost. They are possible on for amino acids occupying two doublet codons (L and R or S depending on the scenario).

2. Mutations to another amino acid in the same chemical group have cost = 1. E.g., H to K are both bases.

3. Mutations across chemical groups have cost = 2.

4. C is a polar amino. Mutations to or from another polar amino acid have cost = 2.5. (If the CMMH is assumed to be absent, cost = 1.)

5. Mutations to or from C with amino acids that are not polar have cost = 3. (If the CMMH is assumed to be absent, cost = 2.)

6. Mutations to Stop by amino acids other than C, cost = 3. The Stop does not mutate. (If the CMMH is assumed to be absent, cost = 2 as if mutating to different chemical group.)

7. Mutation from C to Stop, cost = 4. (If the CMMH is assumed to be absent, cost = 2.)

**Table S3: Table of mutation costs**. Each row represents an amino acid and its 14 possible alternative mutated states. The Stop codon (X) is assumed not to mutate. The numbers represent the mutation costs (see above) associated with coding for a different amino acid or for a Stop signal. These costs are independent of the codons involved.


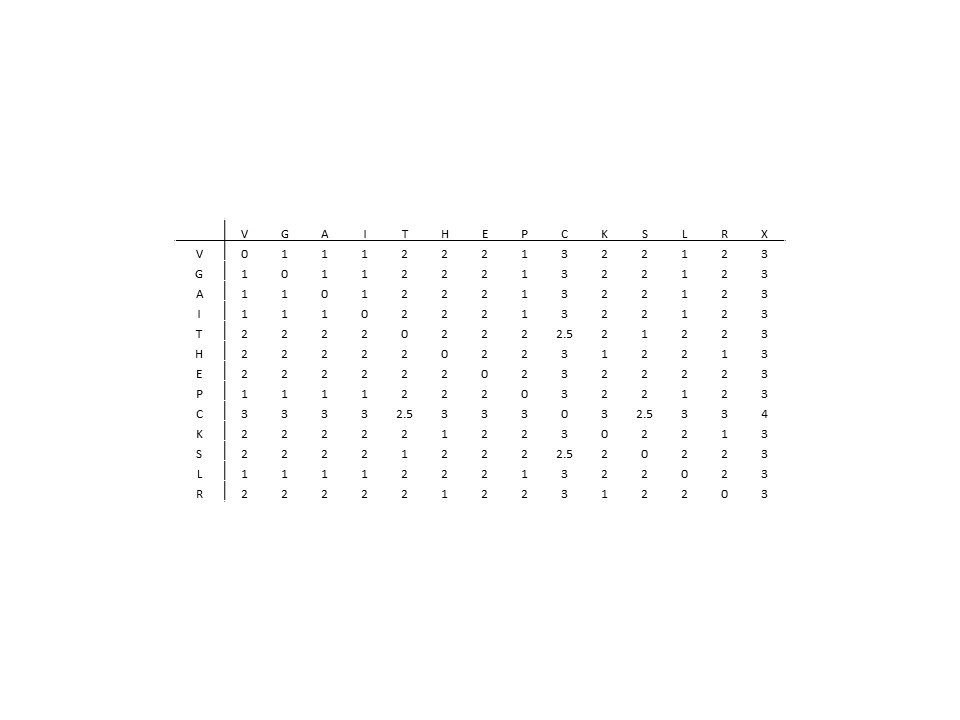


*D. Generating a genome-level mutation distance value*.

We calculate the network-wide mutation distances for the proposed doublet genetic code (Fig. 1). The calculations are shown in Table S4. The values for amino acids at each codon are given in Figure 2.

**Table S4**. **Network-wide mutational distance scores for proposed antecedent doublet genetic codes**. The mutation likelihoods (for low and high scenarios of the ratios of transitions to transversions likelihoods) and codon location probabilities are derived from Table S2. The first column presents the order in which amino acids were recruited into the genetic code, and at which codon location. Columns 2-16 give the amino acids (or Stop: X) at all the mutational distances of all the other codons in the network relative to the codon in Column 1. The costs of mutating to the amino acid associated with the mutated codon are derived from the pairwise relationships in Table S3. Codon AG could be associated with either R or S (see Methods in text). The mutational score of the predicted antecedent doublet network is calculated as the sum of all table cells (which individually are: cost x likelihood x probability), under the varying assumptions that R or S is associated with codon AG and the transitions are twice or ten times as likely as transversions.

*
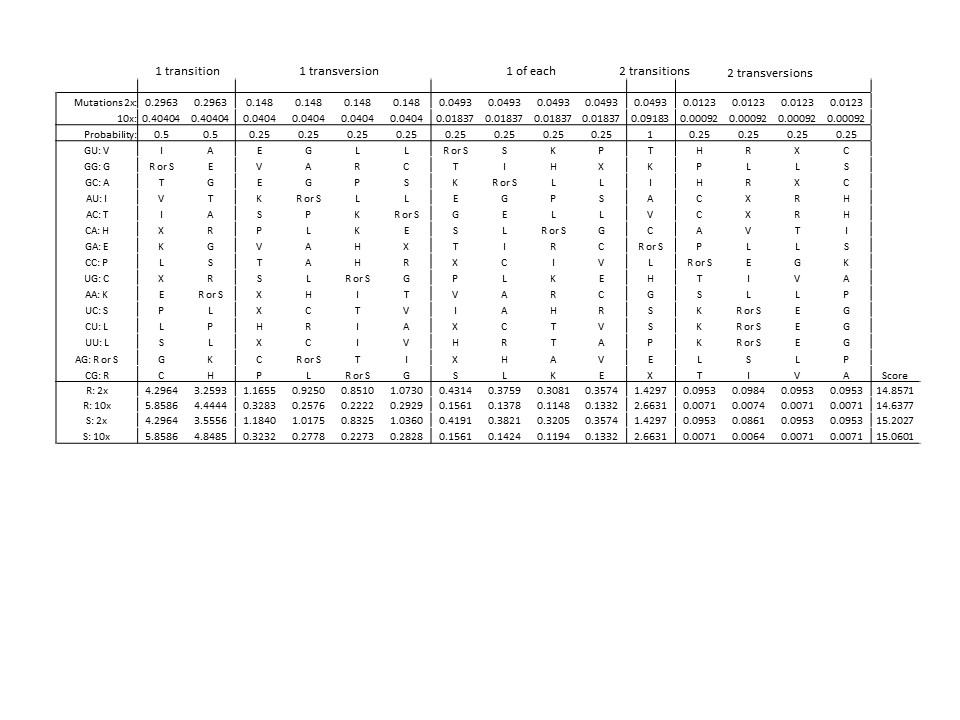
*

*E. Running the randomized simulations*.

At the beginning of each replicate, the 12 amino acids (I to R) are randomly redistributed across the open codons (i.e., randomizing their order in Table S4, Column 1). This will also change the amino acid order across all the rows and Columns 2-16. The values from Tables S2 and S3 remain unchanged. The simulations are replicated 25,000 times in R. See Supplement: R code.

**Supplement Figures.**

**
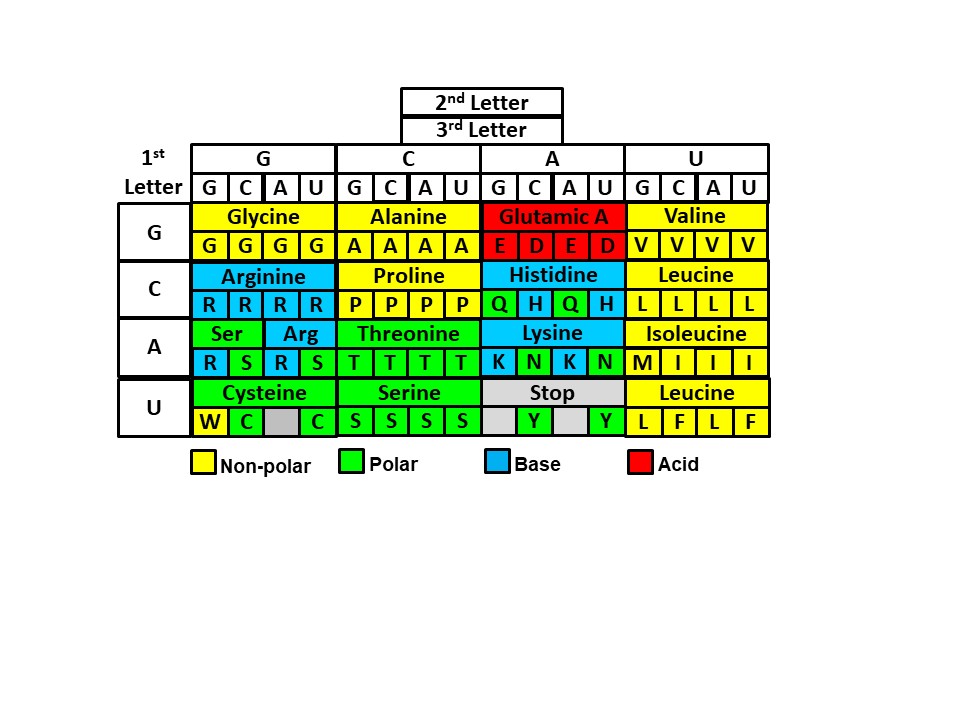
**

**Supplement Figure S1. The present-day genetic code**. The ‘Four-Column’ hypothesis (Higgs 2009) proposes that the transition from a 4-codon singlet code to a doublet code began with making G in the 1^st^ letter of a triplet codon informational (i.e., the top row of the figure: GG = Glycine, GC = alanine, GA = aspartic or glutamic acid, and GU = valine). We propose instead that rather than GA, the fourth doublet was UA and coded as a Stop signal. We use the current occupation at the 1^st^ and 2^nd^ letter and entry order (Wehbi et al. 2024) to assign amino acids and Stop to doublets (NNX) in a proposed antecedent evolving code (Fig. 1). The only non-definitive assignment is for codon AGX, which could be either R or S. Colors define the chemical category of the amino acid.

**Figure S2 A-D**. **Frequency distributions of randomized codon / amino acid pairings**. The data are plotted as proportions (network-wide mutational values / maximum network-wide value). Panels A-D are generated by the CMMH assumptions. Panels E-H are generated under assumptions that cysteine is no different in cost than other polar amino acids and no Stop codon is present. Either arginine or serine is present at two codons and transitions are twice or ten times more likely than transverse mutations. Red bars are wherein the network-wide mutations cost scores of proposed antecedent doublet code (Fig. 1) falls. The p values are the proportion of randomly simulated networks that have lower scores. Given the specific prediction of minimizing network-wide mutation costs, a one-tailed criterion for significance is used (p < 0.1). The Akaike Information Criterion (AIC) to a random distribution for codon to amino acid pairing is always lower (a better fit) when Stops are absent and cysteine is not unique (panels E-G), than when such higher mutation costs are present (panels A-D, respectively).


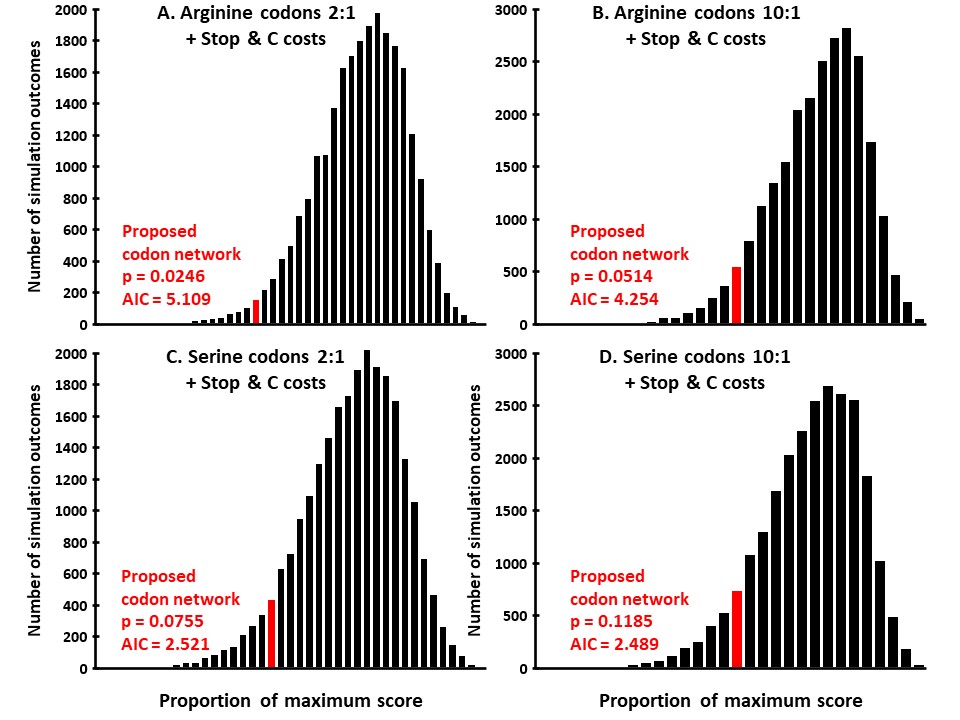


**Figure S2 (cont.) E-H**. **Frequency distributions of randomized codon / amino acid pairings**.


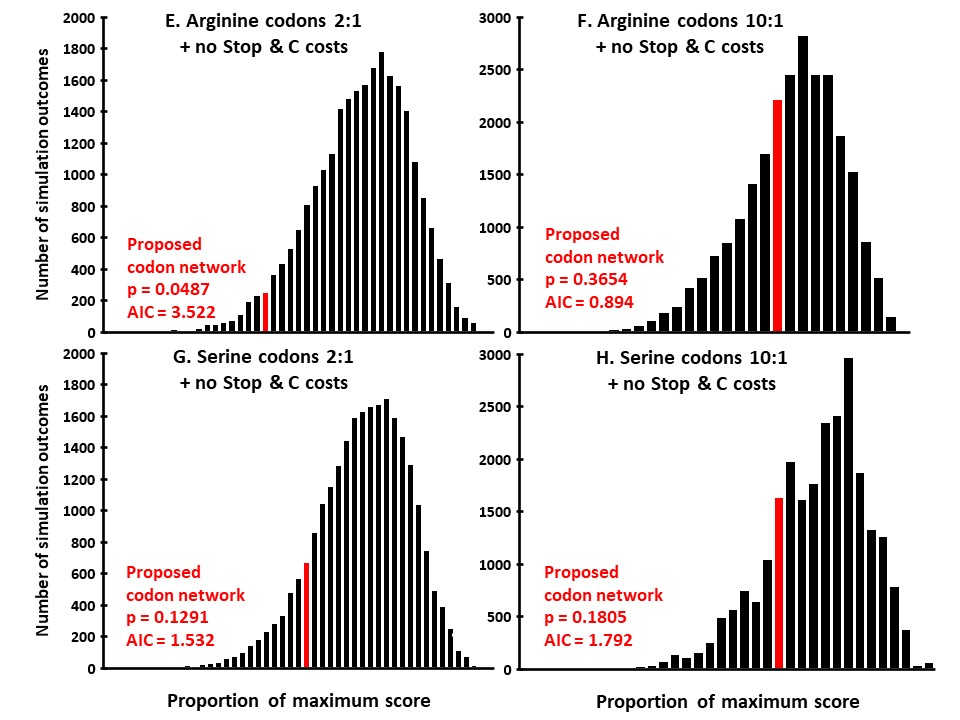


**Figure S3. Optimal codon networks that would minimize catastrophic mutations**. In (A) arginine (a base amino acid) would occupy two of the codons, in (B) serine (a polar amino acid) occupies two codons. Box colors refer to amino acid chemical properties as in Figure S1. Blank boxes indicate chemical group, but are indifferent for the exact amino acid. In both, network-wide mutational values are minimized by the clumping of amino acids by chemical grouping.


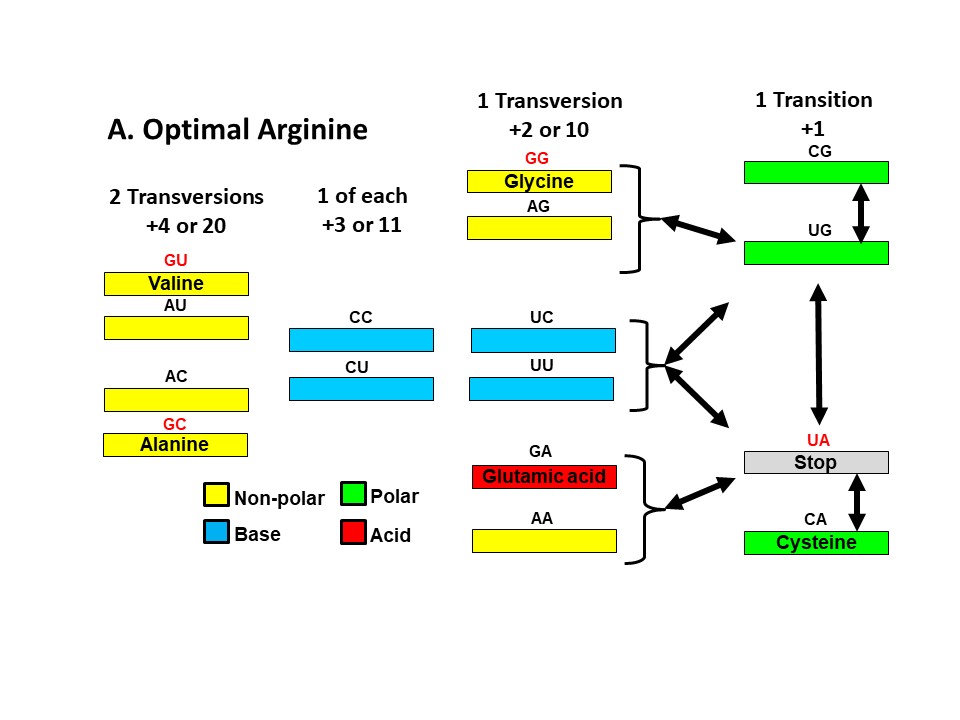


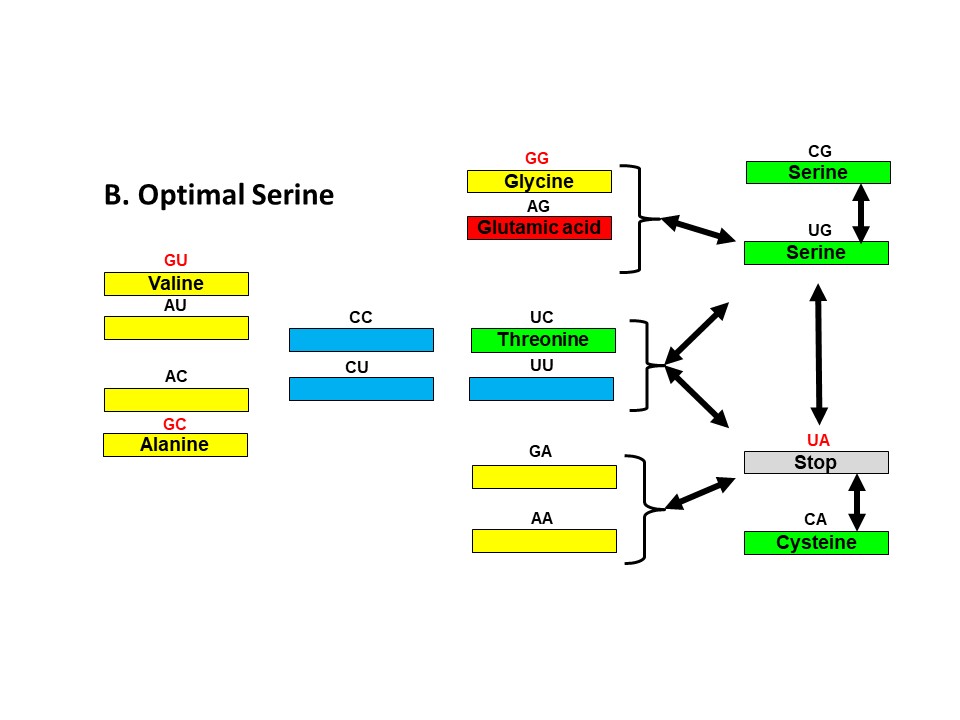


**Figure S4. Predicting the codon placement of cysteine**. In A & B, cysteine has a greater mutational cost than the other polar amino acids (threonine and serine). When randomly simulated with cysteine at UGX and serine at AGX, 7.55% and 11.85% of codon arrangements have lower network-wide mutation costs than does a proposed antecedent code (Supplement Fig. 2C&D). In the subsets of those ‘better’ arrangements, cysteine is disproportionally more likely to be associated with either UGX or CAX (both are one transition mutation from UAX Stop), relative to the 10 other possible codon locations. Also, as the mutation distance to Stop increases across codons (ranked on the x-axis by mutation distance score), the probability of a cysteine being paired with that codon decreases. In contrast in C & D, cysteine is assumed to have the same costs as the two other polar amino acids. Random simulations under these conditions, predicts no preferential codon usage in the ‘better’ arrangements for any of the three polar amino acids. Cysteine is not predicted to be more likely associated with either UGX or CAX. Increasing MDS of a codon does not affect pairings to cysteine. X-axes rank codons by increasing MDS cost. In panels A and C, transverse mutations are more likely; in B and D they are less likely.

**
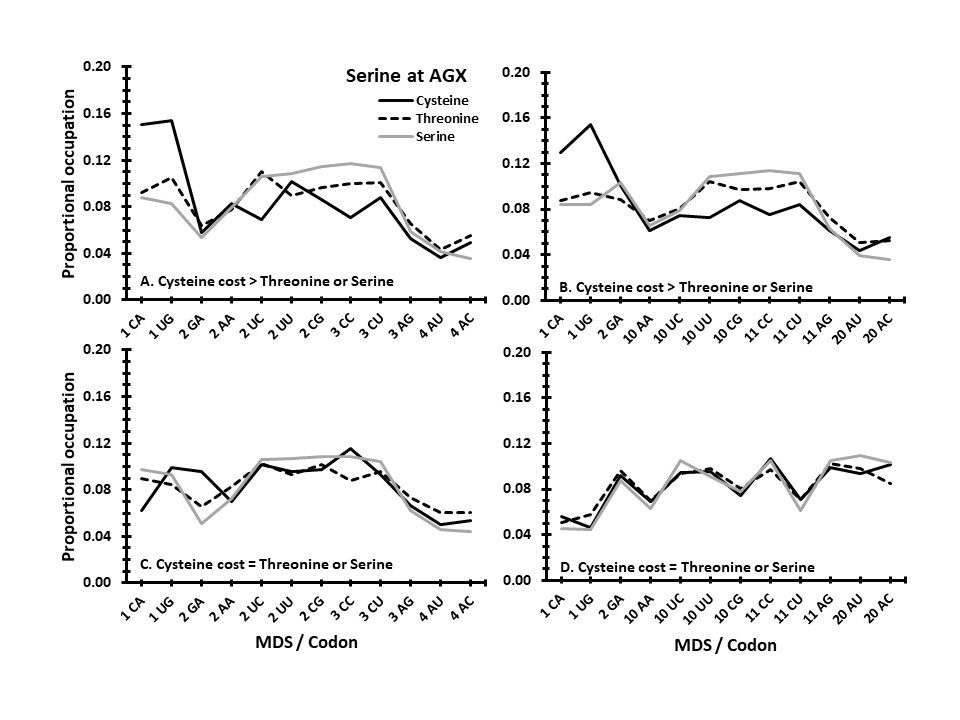
**

**Supplement: R code.**

The code for running the simulated mutational distance values in the antecedent doublet code with amino acid codon locations randomized 250,000 times. The simulations were replicated with arginine or serine at two codons and with transition mutations being twice or ten times more likely than transverse (= 4 different scenarios). The same randomizing series of distributions was used to compare 2x versus 10x.

library(readxl)

library(doParallel)

library(foreach)

library(doRNG)

# Read these once outside the loop

load("ReplaceMatrix_and_CostKey.RData")

# Set up parallel backend

num_cores <- parallel::detectCores() - 1

cl <- makeCluster(num_cores)

registerDoParallel(cl)

#seed for repeatability

#registerDoSEQ()

#registerDoRNG(seed = 1234)

combined_df_all <- foreach(simuNum = 1:25000, .combine = rbind, .packages = c("readxl")) %dopar% {

# Define codon-amino acid mapping

df <- data.frame(

Col1 = c("AG", "AC", "AA", "AU", "GA", "CA", "CC", "CG", "CU", "UC", "UG", "UU"),

Col2 = c("Arginine", "Threonine", "Lysine", "Isoleucine", "Glutamic acid",

"Histidine", "Proline", "Arginine", "Leucine", "Serine",

"Cysteine", "Leucine"), stringsAsFactors = FALSE)

# Shuffle Col2

df$Col2 <- sample(df$Col2)

df2 <- data.frame(Col1 = c("GG", "GC", "GU"), Col2 = c("Glycine", "Alanine", "Valine"), stringsAsFactors = FALSE)

dfStop <- data.frame(Col1 = c("UA"), Col2 = c("STOP"), stringsAsFactors = FALSE)

df3 <- rbind(df2, df, dfStop)

# Replace codons in ReplaceMatrix

ReplaceMatrix <- apply(ReplaceMatrix_orig, c(1, 2), function(x) {

match_idx <- match(x, df3$Col1)

if (!is.na(match_idx)) df3$Col2[match_idx] else x })

cost_matrix <- ReplaceMatrix

for (networkNum in 1:nrow(ReplaceMatrix)) {

currentCodon <- df3$Col2[networkNum]

for (i in 1:ncol(ReplaceMatrix)) { cost_matrix[networkNum, i] <- cost_key[currentCodon, ReplaceMatrix[networkNum, i]] }}

cost_matrix <- as.matrix(cost_matrix)

storage.mode(cost_matrix) <- "numeric"

cost_matrix2<-cost_matrix

cost_matrix[, 1:2] <- cost_matrix[, 1:2] * 0.2963 * 0.5

cost_matrix[, 3:6] <- cost_matrix[, 3:6] * 0.148 * 0.25

cost_matrix[, 7:10] <- cost_matrix[, 7:10] * 0.0493 * 0.25

cost_matrix[, 11] <- cost_matrix[, 11] * 0.0493

cost_matrix[, 12:15] <- cost_matrix[, 12:15] * 0.0123 * 0.25

cost_matrix2[, 1:2] <- cost_matrix2[, 1:2] * 0.40404 * 0.5

cost_matrix2[, 3:6] <- cost_matrix2[, 3:6] * 0.04040 * 0.25

cost_matrix2[, 7:10] <- cost_matrix2[, 7:10] * 0.1837 * 0.25

cost_matrix2[, 11] <- cost_matrix2[, 11] * 0.09183

cost_matrix2[, 12:15] <- cost_matrix2[, 12:15] * 0.00092 * 0.25

row_sums <- rowSums(cost_matrix)

row_sums2 <- rowSums(cost_matrix2)

df3_trimmed <- df3[1:nrow(cost_matrix), ]

combined <- cbind(df3_trimmed, RowSum = row_sums, RowSumNew = row_sums2)

# Create new column names like "1_A", "2_B", etc.

numbered_names <- paste0(seq_along(combined$Col1), "_", combined$Col1)

# Create named list from Col2 values

cols_list <- setNames(as.list(combined$Col2), numbered_names)

# Build final data frame

result <- data.frame(

iteration = simuNum,

CostSum = sum(combined$RowSum),

CostSumNew = sum(combined$RowSumNew),

stringsAsFactors = FALSE )

# Add transposed Col2 values with numbered Col1 names

cbind(result, as.data.frame(cols_list, stringsAsFactors = FALSE))}

write.csv(combined_df_all, "ArginineArginine_Run.csv")

combined_df_all_SerineSerine <- foreach(simuNum = 1:25000, .combine = rbind, .packages = c("readxl")) %dopar% {

# Define codon-amino acid mapping

df <- data.frame(

Col1 = c("AG", "AC", "AA", "AU", "GA", "CA", "CC", "CG", "CU", "UC", "UG", "UU"),

Col2 = c("Arginine", "Threonine", "Lysine", "Isoleucine", "Glutamic acid",

"Histidine", "Proline", "Serine", "Leucine", "Serine",

"Cysteine", "Leucine"), stringsAsFactors = FALSE)

# Shuffle Col2

df$Col2 <- sample(df$Col2)

df2 <- data.frame(

Col1 = c("GG", "GC", "GU"),

Col2 = c("Glycine", "Alanine", "Valine"),

stringsAsFactors = FALSE )

dfStop <- data.frame(Col1 = c("UA"), Col2 = c("STOP"), stringsAsFactors = FALSE)

df3 <- rbind(df2, df, dfStop)

# Replace codons in ReplaceMatrix

ReplaceMatrix <- apply(ReplaceMatrix_orig, c(1, 2), function(x) {

match_idx <- match(x, df3$Col1)

if (!is.na(match_idx)) df3$Col2[match_idx] else x })

cost_matrix <- ReplaceMatrix

for (networkNum in 1:nrow(ReplaceMatrix)) {

currentCodon <- df3$Col2[networkNum]

for (i in 1:ncol(ReplaceMatrix)) { cost_matrix[networkNum, i] <- cost_key[currentCodon, ReplaceMatrix[networkNum, i]] } }

cost_matrix <- as.matrix(cost_matrix)

storage.mode(cost_matrix) <- "numeric"

cost_matrix2<-cost_matrix

cost_matrix[, 1:2] <- cost_matrix[, 1:2] * 0.2963 * 0.5

cost_matrix[, 3:6] <- cost_matrix[, 3:6] * 0.148 * 0.25

cost_matrix[, 7:10] <- cost_matrix[, 7:10] * 0.0493 * 0.25

cost_matrix[, 11] <- cost_matrix[, 11] * 0.0493

cost_matrix[, 12:15] <- cost_matrix[, 12:15] * 0.0123 * 0.25

cost_matrix2[, 1:2] <- cost_matrix2[, 1:2] * 0.40404 * 0.5

cost_matrix2[, 3:6] <- cost_matrix2[, 3:6] * 0.04040 * 0.25

cost_matrix2[, 7:10] <- cost_matrix2[, 7:10] * 0.1837 * 0.25

cost_matrix2[, 11] <- cost_matrix2[, 11] * 0.09183

cost_matrix2[, 12:15] <- cost_matrix2[, 12:15] * 0.00092 * 0.25

row_sums <- rowSums(cost_matrix)

row_sums2 <- rowSums(cost_matrix2)

df3_trimmed <- df3[1:nrow(cost_matrix), ]

combined <- cbind(df3_trimmed, RowSum = row_sums, RowSumNew = row_sums2)

# Create new column names like "1_A", "2_B", etc.

numbered_names <- paste0(seq_along(combined$Col1), "_", combined$Col1)

# Create named list from Col2 values

cols_list <- setNames(as.list(combined$Col2), numbered_names)

# Build final data frame

result <- data.frame(

iteration = simuNum,

CostSum = sum(combined$RowSum),

CostSumNew = sum(combined$RowSumNew),

stringsAsFactors = FALSE)

# Add transposed Col2 values with numbered Col1 names

cbind(result, as.data.frame(cols_list, stringsAsFactors = FALSE))}

write.csv(combined_df_all_SerineSerine, "SerineSerine_Run.csv")

# Stop the cluster

stopCluster(cl)

registerDoSEQ()
